# Supplementary material for: Upstream interventions to promote oral health and reduce socioeconomic oral health inequalities: a scoping review protocol
Source: BMJ Open. 2022 Jun 23;12(6):e059441. doi: 10.1136/bmjopen-2021-059441 (PMC9226867; doi:10.1136/bmjopen-2021-059441)

APPENDIX

Appendix A: Search strategy for a scoping review protocol on upstream interventions to promote oral health and reduce socio-economic oral health inequalities.

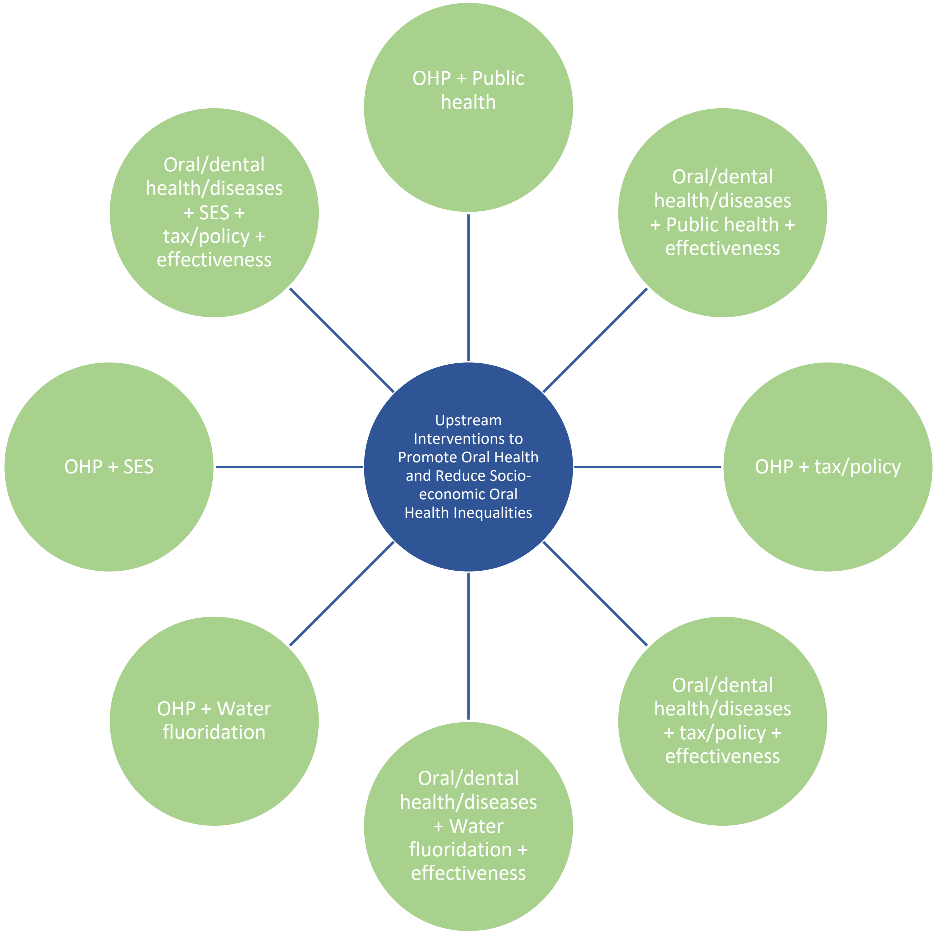

Supplement: Supplementary data [file bmjopen-2021-059441supp001.pdf]
