# Supplementary material for: Upstream interventions to promote oral health and reduce socioeconomic oral health inequalities: a scoping review protocol
Source: BMJ Open. 2022 Jun 23;12(6):e059441. doi: 10.1136/bmjopen-2021-059441 (PMC9226867; doi:10.1136/bmjopen-2021-059441)
Supplement: Author's manuscript [file bmjopen-2021-059441.draft_revisions.pdf]

BMJ Open

BMJ Open is committed to open peer review. As part of this commitment we make the peer review history of every article we publish publicly available.

When an article is published we post the peer reviewers' comments and the authors' responses online. We also post the versions of the paper that were used during peer review. These are the versions that the peer review comments apply to.

The versions of the paper that follow are the versions that were submitted during the peer review process. They are not the versions of record or the final published versions. They should not be cited or distributed as the published version of this manuscript.

BMJ Open is an open access journal and the full, final, typeset and author-corrected version of record of the manuscript is available on our site with no access controls, subscription charges or pay-per-view fees (http://bmjopen.bmj.com).

If you have any questions on BMJ Open's open peer review process please email info.bmjopen@bmj.com

BMJ Open

Upstream Interventions to Promote Oral Health and Reduce Socio-economic Oral Health Inequalities: A Scoping Review Protocol

Journal:	*BMJ Open*
Manuscript ID	bmjopen-2021-059441
Article Type:	Protocol
Date Submitted by the Author:	30-Nov-2021
Complete List of Authors:	Dawson, Eleanor; University College London, Department of Epidemiology and Public Health<br>Stennett, Michelle; University College London, Department of Epidemiology and Public Health<br>Daly, Blánaid; Trinity College Dublin, Division of Public and Child Dental Health<br>Macpherson, Lorna; University of Glasgow, Dental Public Health Section, School of Medicine, Dentistry and Nursing<br>Cannon, Paul; University of Glasgow, University Library<br>Watt, Richard; University College London, Department of Epidemiology and Public Health
Keywords:	PUBLIC HEALTH, QUALITATIVE RESEARCH, Health policy < HEALTH SERVICES ADMINISTRATION & MANAGEMENT

SCHOLARONE™
Manuscripts

Upstream Interventions to Promote Oral Health and Reduce Socio-economic Oral Health Inequalities: A Scoping Review Protocol

Authors
Eleanor R. Dawson[1], Michelle Stennett[1], Professor Blánaid Daly[2], Professor Lorna Macpherson[3], Paul Cannon[4] and Professor Richard G Watt[1]

1. Department of Epidemiology and Public Health, University College London, London, UK

2. Division of Public and Child Dental Health, Dublin Dental University Hospital and Dental School, University of Dublin, Trinity College Dublin, Dublin, Ireland

3. Dental Public Health Section, School of Medicine, Dentistry and Nursing, University of Glasgow, Glasgow, UK

4. University Library, Hillhead Street, University of Glasgow, G12 8QE, UK

Corresponding author: Eleanor R. Dawson, Department of Epidemiology and Public Health, University College London, London, UK e.dawson@ucl.ac.uk

Word Count: 2124

For peer review only

For peer review only - http://bmjopen.bmj.com/site/about/guidelines.xhtml

ABSTRACT

Introduction: Improving oral health and reducing oral health inequalities is an important global health priority. 'Upstream interventions' are a vital part of the collective effort to reduce oral disease burdens, however it is a rather nebulous term. Furthermore, there is little evidence on the effectiveness, impact and sustainability of upstream interventions that have previously targeted oral health and wider public health measures that impact on oral health. The aim of this scoping review is to systematically map and synthesize evidence on the effectiveness, impact and sustainability of upstream interventions on population oral health and reducing socio-economic oral health inequalities.

Methods and analysis: This scoping review will be conducted in accordance with the Joanna Briggs Institute (JBI) methodology and the Preferred Reporting Items for Systematic Reviews and Meta-Analyses Extension for Scoping Reviews (PRISMA-ScR) checklist. Literature published in any language will be included with no restrictions on publication dates. A detailed search strategy will be used to conduct a comprehensive search of electronic databases; Scopus, Embase and MEDLINE, PsycINFO and CINAHL, ASSIA and Cochrane Database of Systematic Reviews. A search of grey literature will also be completed to identify relevant dissertations, governmental reports and evaluations of implemented policies. Identification and extraction of data will be performed by two pairs of reviewers. Oversight and feedback will be provided by an independent expert advisory group.

Ethics and dissemination: This study will only use published information and therefore does not require an ethics review. The scoping review protocol has been registered with the Open Science Framework. The final report will be circulated and disseminated through publication and feed into the work of the ongoing Lancet Commission on Oral Health. Due to the policy relevance of this work, discussions will take place with key stakeholders regarding the implications of the findings for future policy development.

Strengths and limitations of this study

- This is the first scoping review to map the evidence about the effectiveness of upstream interventions on population oral health and socio-economic oral health inequalities
- This review will include literature published in any language and also the wider grey literature.
- This review will assess the broad evidence base for upstream interventions on oral health outcomes and socio-economic inequalities but will not undertake a detailed evaluation of interventions
- The results of this review will inform oral health policy and identify gaps in research

For peer review only - http://bmjopen.bmj.com/site/about/guidelines.xhtml

INTRODUCTION

Oral diseases are a highly prevalent global public health problem, affecting approximately 3.5 billion people, which equates to almost half of the world's population (1). Oral diseases (such as dental caries, periodontal disease, and oral cancer) are largely preventable and in recent decades some improvements in oral health have been observed predominantly in high-income countries (2). However dental caries in many low- and middle-income countries appears to be on the increase, linked to economic development and increasing availability and consumption of free sugars (3). Even in high-income countries with lower disease burdens, dental services are largely treatment oriented without fully addressing the underlying causes of oral diseases (4).

Poor oral health may lead to profound impacts on individuals and wider society. Pain or sepsis (4) lower self-esteem (5), reduced school attendance and poorer educational performance (6), poorer quality of life (7) and reduced work productivity (8) are all common impacts of oral diseases. There are also significant economic costs associated with dental treatment and lost productivity, amounting to approximately $544 billion globally in 2015 (9). Oral diseases disproportionately affect poorer, socially disadvantaged and marginalised groups; such differences are deemed to be avoidable, unfair and unjust in modern society (3). Furthermore, stark social gradients exist between socio-economic position and the prevalence and severity of oral conditions (10,11), reflecting similar patterns of inequality seen in general health.

Contemporary concepts regarding the determinants of health now largely acknowledge the underlying influence of structural and societal factors in socially patterning individual health behaviours and ultimately in determining disease levels (12). Indeed, McKinlay (13) originally utilised the metaphor of people being pushed into a flowing river to describe the causes, or upstream 'manufacturers' of ill health in comparison to the downstream effects of illness. However, this metaphor can also be employed to highlight the different intervention opportunities (upstream, midstream or downstream) for public health programmes and policies to improve population health (14).

Downstream interventions (individual/clinical/behavioural measures) have often been implemented to combat oral diseases and have failed to significantly reduce oral health inequalities or achieve sustainable improvements in oral health outcomes (15). For example, individual health education interventions tend to result in limited short-term positive changes in oral health related behaviours and health literacy, with marginal long-term improvements in clinical outcomes (16–18). Moreover, evidence has demonstrated that downstream interventions may adversely result in widening health inequalities between socio-economic groups, as they tend to provide more benefit for groups who are already advantaged (19). This widening of the health inequality gap is often referred to as the 'inverse prevention law' (20) and has been observed with oral health education interventions (21,22).

Upstream interventions are those that seek to address the underlying causative factors that lead to poor health, typically through action on broader structural, political, economic and environmental determinants. The term 'upstream intervention' is often a nebulous phrase to define in practice, as there may be difficulty in identifying broader public health approaches

For peer review only

For peer review only - http://bmjopen.bmj.com/site/about/guidelines.xhtml

as purely ‘upstream’, as well as complexity in accurately defining population coverage and effectiveness of interventions. Overall, upstream interventions attempt to achieve sustained health equity in a population, often outside of the sphere of health care systems (23).

In this scoping review, upstream interventions will be defined as ‘strategies or policies that seek to address structural or environmental factors that impact population oral health and socio-economic oral health inequalities. This will include population level approaches, such as fiscal measures, regulation and legislation’. Such interventions generally aim to tackle broader social determinants (such as education, housing, access to safe drinking water and healthy food), or may create disincentives to engage in harmful health-related behaviours, such as fiscal policies to reduce consumption of sugary drinks (24). These interventions are generally initiated by national governments or policy makers and operate in a top-down manner across whole populations.

A broad consensus now supports the need for a combination of downstream, mid-stream and upstream interventions in order to effectively prevent oral diseases and promote oral health equity (4,25). However, questions remain over the extent of evidence surrounding the effectiveness of upstream interventions in promoting oral health and reducing socio-economic health inequalities. This review will include the following upstream interventions for consideration; the implementation of water fluoridation schemes, regulations and fiscal measures aimed at reducing the consumption of tobacco, alcohol and sugar and interventions aimed at enhancing population welfare through improvement of housing, education and healthcare access. These upstream policies are those most commonly identified in the literature, however, the identification of other upstream interventions during the conduct of this review will also be included.

Although there are a wide range of persistent inequalities in oral health (such as inequalities between and within countries, ethnic differences and poorer outcomes in various vulnerable groups), this scoping review will focus on the impact of upstream interventions on promoting oral health and reducing socio-economic oral health inequalities.

A scoping review has been chosen as the most appropriate form of evidence synthesis for this area of research as it will incorporate a broader scope and less restrictive inclusion criteria than a systematic review (26). The proposed work of this review will be linked to the ongoing Lancet Commission on Oral Health, which includes an oral health inequalities workstream, and the findings will be shared with other key stakeholders regarding the possible implications for policy development.

For peer review only

For peer review only - http://bmjopen.bmj.com/site/about/guidelines.xhtml

METHODS

A preliminary search of SCOPUS, Google Scholar, Cochrane Database of Systematic Reviews and *JBI Evidence Synthesis* was conducted in June 2021. No ongoing or previously published scoping reviews on this topic was identified. Oversight and feedback of the methodology will be provided by an independent expert advisory group.

Scoping review questions

The aim of this scoping review is to identify the current literature that documents implemented upstream interventions and their effect on promoting oral health and reducing socio-economic oral health inequalities. It will aim to address the following questions:

- What upstream interventions have been implemented to specifically target a reduction in socio-economic oral health inequalities and promote oral health?
- What relevant and related public health upstream interventions eg tobacco control policies have been implemented to reduce socio-economic health inequalities and promote health?
- Which upstream interventions have had an impact, are effective and are sustainable in reducing socio-economic health inequalities and improving population oral health?

Search Strategy

The search strategy will aim to locate both published studies and relevant grey literature. An initial limited search of SCOPUS and PubMed was undertaken to identify articles relevant to the topic. The text words contained in the titles and abstracts of relevant articles and the index terms used to describe the articles will be used to develop a full search strategy on: Scopus, Embase and MEDLINE (via Ovid), PsycINFO and CINAHL (via EBSCOhost), ASSIA (via ProQuest) and Cochrane Database of Systematic Reviews (via the Cochrane Library). Sources of grey literature to be searched include NTIS, NICE Evidence Search, TRIP, EThOS and WorldCat. The websites of relevant healthcare organisations will also be searched, for example the World Health Organization (WHO), Public Health England (PHE), World Bank and The Health Foundation. Relevant national or state level government policy documents will be examined. A manual search of bibliographies of selected articles will be undertaken by the research team to extract any relevant literature or identify grey literature.

Given the difficulty defining upstream interventions, a multi-stranded search method (27) will be employed, with each strand representing an intervention of interest, namely: health inequalities, health disparities, health inequities, oral health promotion, tobacco control, sugar tax, upstream action, food advertising, food labelling and public health (Appendix A). The dental and oral health elements of the search will be adapted from the strategies employed by Waldron et al. (28) and Arora et al. (29). The sugar tax strand of the search will use the strategies of Pfinder et al. (30) and the equity-focused strand will use the strategies of Prady et al. (31).

For peer review only

Study selection

Inclusion criteria

-Participants and Context

Only studies that include population-wide polices or studies that target certain population groups will be included in this review. This includes populations that have been specifically targeted for the intervention or the general population. There is no minimum or maximum number for the size of the population group or age restrictions. The review will be limited to literature that includes implemented upstream interventions. The review will include all global settings where upstream interventions have been brought in to directly impact population oral health.

-Patient and public involvement

There will be no patient involvement.

-Concepts and Definitions

The primary concept in this review is 'upstream interventions.' There is no unified definition of this term, but this scoping review has utilised a definition collating input from a variety of previous publications (13,14,15,25). Upstream interventions will be defined as 'strategies or policies that seek to address structural or environmental factors that impact population oral health and socio-economic oral health inequalities. This will include population level approaches, such as fiscal measures, regulation and legislation'.

Oral health inequalities are unequal and unfair differences in oral health outcomes across different groups in society (4). Such differences often disproportionally affect poorer and socially disadvantaged members of society. The association between poor oral health and socioeconomic status is well documented and is prevalent throughout the life course and across populations regardless of national income status.

-Sources and types of studies

This scoping review will include peer reviewed published journal articles, grey literature and relevant government reports and policy documents. There is no limit on date and no language restrictions will be applied to the search. This review will consider a variety of study designs, including experimental, quasi-experimental, before and after studies and observational studies (including cohort studies, case-control studies and cross-sectional studies). In addition, systematic reviews will be included in this review, providing they meet the inclusion criteria. The review will also include other relevant public health policies and interventions, for example, sugar and tobacco control measures.

Exclusion criteria

Studies reporting on clinical interventions delivered in clinical settings and restricted to specific patient populations will be excluded from this review. Studies only reporting on oral health knowledge and attitudes and reviews of websites or industry documents will also not be included.

For peer review only

Data extraction

The final data from the search strategy will deposited into the database Rayyan (http://rayyan.qcri.org) where two pairs of reviewers will independently screen the titles and abstracts of each citation using the inclusion and exclusion criteria to identify eligible articles for full review. Full texts will be extracted into a data extraction form developed by the research team in Microsoft Word. The data extracted will include the following details: study characteristics (first author, publication year, country, published language, population size and characteristics, duration and setting) type of upstream intervention, outcomes and key findings relevant to the review question. The data extraction tool will be modified and revised as necessary during the process of extracting data from each included evidence source. If appropriate, authors of papers will be contacted to request missing or additional data, where required.

Collating, summarising and reporting the data

All of the relevant screened articles will be stored in the reference manager, Zotero (2020/ version 5.0.88). The summarised characteristics of the extracted studies will be presented visually in tabular form due to large number of studies that will be found. Results from the selected studies will also be presented in a narrative format which will relate directly to the review questions and aims. The final report will be presented in accordance with the JBI methodology (26) and the Preferred Reporting Items for Systematic Reviews and Meta-Analyses Extension for Scoping Reviews (PRISMA-ScR) checklist (32).

ETHICS AND DISSEMINATION

The final protocol has been submitted for registration with Open Science Framework. A detailed final report will be produced on the review methods, results and recommendations following completion of the scoping review. A final paper presenting the review results will be also prepared for publication in a peer reviewed journal on completion of the review. The findings from this review will feed directly into the Lancet Commission on Oral Health and will be highlighted in the Commission Report depending upon the final structure of the report. Due to the strong policy relevance of this work, discussions will take place with the funder (The Borrow Foundation) on completion of the review to decide whether to hold a dissemination workshop with key policy stakeholders regarding the relevance of the findings for future policy development. The findings from this review may also be presented through other relevant forums, such as international conferences or scientific meetings.

CONCLUSION

The knowledge generated from this scoping review will have short term impacts in assisting the development of a new research agenda, as well as longer term impacts regarding the development and implementation of policy recommendations, resulting in an improvement of population oral health.

For peer review only - http://bmjopen.bmj.com/site/about/guidelines.xhtml

CONTRIBUTIONS

RGW, MS, BD and LM conceived the idea for the review. RGW, ED, MS, BD, PC and LM all contributed to the development of the protocol. ED, RGW and MS drafted the original manuscript. RGW, LM, PC and BD edited and revised the manuscript. ED conducted the preliminary search and PC provided expertise on the final search strategy. All authors edited and approved the final text prior to submission for publication.

ACKNOWLEDGMENTS

The research team would like to thank the expert advisory group for their input into the development of the protocol and the scoping review design and methodology. The advisory group is composed of, Professor Roger Keller Celeste (UFRGS, Brazil), Dr Manu Raj Mathur (University of Liverpool, UK/Public Health Foundation of India), Dr. Mary Jane McCallum (Canada), Professor Robert J. Weyant (University of Pittsburg, USA), Dr Jenny Godson and Dr Semina Makhani (Public Health England). The research team would also like to thank Sana Daniyal and Afshan Mirza for their support in undertaking the initial screening and reviewing of eligible papers.

FUNDING

This work was supported by The Borrow Foundation (UK Charity No: 1060308), grant number not applicable. The Borrow Foundation has no further role in the conduct of the review other than providing the designated funding.

COMPETING INTERESTS

None declared.

For peer review only

REFERENCES

1. Kassebaum NJ, Smith AGC, Bernabé E, et al. Global, Regional, and National Prevalence, Incidence, and Disability-Adjusted Life Years for Oral Conditions for 195 Countries, 1990-2015: A Systematic Analysis for the Global Burden of Diseases, Injuries, and Risk Factors. *J Dent Res* 2017;96(4):380-387.

2. GBD 2017 Oral Disorders Collaborators, Bernabe E, Marcenes W, Hernandez CR, et al. Global, Regional, and National Levels and Trends in Burden of Oral Conditions from 1990 to 2017: A Systematic Analysis for the Global Burden of Disease 2017 Study. *J Dent Res* 2020;99(4):362–73.

3. Watt RG, Daly B, Allison P, et al. Ending the neglect of global oral health: time for radical action. *The Lancet* 2019;394(10194):261–72.

4. Peres MA, Macpherson LMD, Weyant RJ, et al. Oral diseases: a global public health challenge. *The Lancet* 2019;20;394(10194):249–60.

5. Kaur P. Impact of Dental Disorders and its Influence on Self Esteem Levels among Adolescents. *J Clin Diagn Res* 2017. Available from: http://jcdr.net/article_fulltext.asp?issn=0973-709x&year=2017&volume=11&issue=4&page=ZC05&issn=0973-709x&id=9515

6. Rebelo MAB, Vieira JMR, Pereira JV, et al. Does oral health influence school performance and school attendance? A systematic review and meta-analysis. *Int J Paediatr Dent* 2019;29(2):138–48.

7. Haag DG, Peres KG, Balasubramanian M, et al. Oral Conditions and Health-Related Quality of Life: A Systematic Review. *J Dent Res* 2017;96(8):864–74.

8. Kelekar U. Hours Lost to Planned and Unplanned Dental Visits Among US Adults. *Prev Chronic Dis* 2018. Available from: https://www.cdc.gov/pcd/issues/2018/17_0225.htm

9. Righolt AJ, Jevdjevic M, Marcenes W, et al. Global, Regional, and Country-Level Economic Impacts of Dental Diseases in 2015. *J Dent Res* 2018;97(5):501–7.

10. Borrell LN, Crawford ND. Socioeconomic position indicators and periodontitis: examining the evidence. *Periodontol 2000* 2012;58(1):69–83.

11. Schwendicke F, Dörfer CE, Schlattmann P, et al. Socioeconomic inequality and caries: a systematic review and meta-analysis. *J Dent Res* 2015;94(1):10–8.

12. Solar O, Irwin A. A Conceptual Framework for Action on the Social Determinants of Health. WHO; 2010.

13. McKinlay J. A case for refocusing upstream: the political economy of illness. In: Applying behavioral science to cardiovascular risk. Seattle: American Heart Association 1975:7–18.

14. McMahon NE. Framing action to reduce health inequalities: what is argued for through use of the 'upstream–downstream' metaphor? *J Public Health* 2021; dab157, https://doi.org/10.1093/pubmed/fdab157

15. Watt RG. From victim blaming to upstream action: tackling the social determinants of oral health inequalities. *Community Dent Oral Epidemiol* 2007;35(1):1–11.

16. Sprod AJ, Anderson R, Treasure ET. Effective oral health promotion: literature review. Database of Abstracts of Reviews of Effects (DARE): Quality-assessed Reviews. UK: Centre for Reviews and Dissemination 1996. Available from: https://www.ncbi.nlm.nih.gov/books/NBK66717/

For peer review only

17. Kay EJ, Locker D. Is dental health education effective? A systematic review of current evidence. *Community Dent Oral Epidemiol* 1996;24(4):231–5.

18. Watt RG, Marinho VC. Does oral health promotion improve oral hygiene and gingival health? *Periodontol 2000* 2005;37:35–47.

19. Lorenc T, Petticrew M, Welch V, et al. What types of interventions generate inequalities? Evidence from systematic reviews. *J Epidemiol Community Health* 2013;67(2):190–3.

20. Gordon D, Shaw M, Dorling D, et al. Inequalities in health: Evidence presented to the independent inquiry into inequalities in health. Bristol: Policy Press 1999.

21. Schou L, Wight C. Does dental health education affect inequalities in dental health? *Community Dent Health* 1994;11(2):97–100.

22. Qadri G, Alkilzy M, Franze M, et al. School-based oral health education increases caries inequalities. *Community Dent Health* 2018;30:35(3):153–9.

23. Williams DR, Costa MV, Odunlami AO, et al. Moving Upstream: How Interventions that Address the Social Determinants of Health can Improve Health and Reduce Disparities. *J Public Health Manag Pract* 2008;14(Suppl):S8-17.

24. WHO. Taxes on sugary drinks: Why do it? Geneva: World Health Organisation; 2017.

25. Baelum V. Dentistry and population approaches for preventing dental diseases. *J Dent*. 2011;39 Suppl 2:S9-19.

26. The Joanna Briggs Institute. Joanna Briggs Institute Reviewers' Manual. The Joanna Briggs Institute 2015.

27. NICE. Developing NICE guidelines: the manual. *National Institute for Health and Care Excellenc*e 2020. Available from: https://www.nice.org.uk/process/pmg20/chapter/identifying-the-evidence-literature-searching-and-evidence-submission

28. Waldron C, Nunn J, Phadraig CMG, et al. Oral hygiene interventions for people with intellectual disabilities. *Cochrane Database Syst Rev* 2019 (5). Available from: https://www.cochranelibrary.com/cdsr/doi/10.1002/14651858.CD012628.pub2/full

29. Arora A, Khattri S, Ismail NM, et al. School dental screening programmes for oral health. *Cochrane Database Syst Rev* 2019;(8). Available from: https://www.cochranelibrary.com/cdsr/doi/10.1002/14651858.CD012595.pub3/full

30. Pfinder M, Heise TL, Boon MH, et al. Taxation of unprocessed sugar or sugar-added foods for reducing their consumption and preventing obesity or other adverse health outcomes. *Cochrane Database Syst Rev* 2020 (4). Available from: https://www.cochranelibrary.com/cdsr/doi/10.1002/14651858.CD012333.pub2/full

31. Prady SL, Uphoff EP, Power M, et al. Development and validation of a search filter to identify equity-focused studies: reducing the number needed to screen. *BMC Med Res Methodo* 2018;12;18(1):106.

32. Tricco AC, Lillie E, Zarin W, et al. PRISMA Extension for Scoping Reviews (PRISMA-ScR): Checklist and Explanation. *Ann Intern Med* 2018;2;169(7):467–73.

For peer review only

For peer review only

For peer review only - http://bmjopen.bmj.com/site/about/guidelines.xhtml

APPENDIX

Appendix A: Search strategy for a scoping review protocol on upstream interventions to promote oral health and reduce socio-economic oral health inequalities.

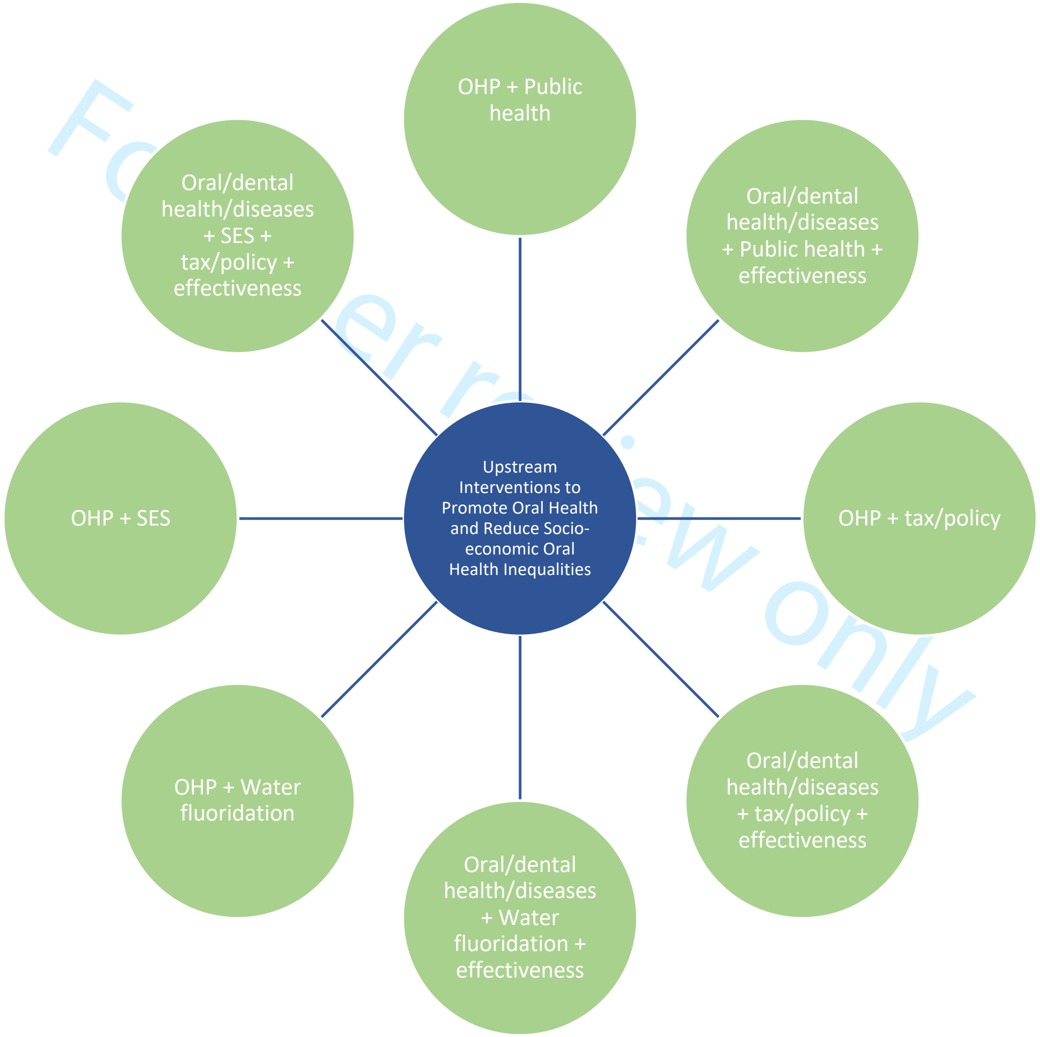

PRISMA-P (Preferred Reporting Items for Systematic review and Meta-Analysis Protocols) 2015 checklist: Upstream Interventions to Promote Oral Health and Reduce Socio-economic Oral Health Inequalities: A Scoping Review Protocol

Section and topic	Item No	Checklist item	Completed
**ADMINISTRATIVE INFORMATION**			
Title:			
Identification	1a	Identify the report as a protocol of a systematic review	X
Update	1b	If the protocol is for an update of a previous systematic review, identify as such	N/A
Registration	2	If registered, provide the name of the registry (such as PROSPERO) and registration number	X
Authors:			
Contact	3a	Provide name, institutional affiliation, e-mail address of all protocol authors; provide physical mailing address of corresponding author	X
Contributions	3b	Describe contributions of protocol authors and identify the guarantor of the review	X
Amendments	4	If the protocol represents an amendment of a previously completed or published protocol, identify as such and list changes; otherwise, state plan for documenting important protocol amendments	N/A
Support:			
Sources	5a	Indicate sources of financial or other support for the review	X
Sponsor	5b	Provide name for the review funder and/or sponsor	X
Role of sponsor or funder	5c	Describe roles of funder(s), sponsor(s), and/or institution(s), if any, in developing the protocol	X
**INTRODUCTION**			
Rationale	6	Describe the rationale for the review in the context of what is already known	X
Objectives	7	Provide an explicit statement of the question(s) the review will address with reference to participants, interventions, comparators, and outcomes (PICO)	X
**METHODS**			
Eligibility	8	Specify the study characteristics (such as PICO, study design, setting, time frame) and report characteristics (such as years	X

For peer review only

criteria		considered, language, publication status) to be used as criteria for eligibility for the review	
Information sources	9	Describe all intended information sources (such as electronic databases, contact with study authors, trial registers or other grey literature sources) with planned dates of coverage	X
Search strategy	10	Present draft of search strategy to be used for at least one electronic database, including planned limits, such that it could be repeated	X
Study records:			
Data management	11a	Describe the mechanism(s) that will be used to manage records and data throughout the review	X
Selection process	11b	State the process that will be used for selecting studies (such as two independent reviewers) through each phase of the review (that is, screening, eligibility and inclusion in meta-analysis)	X
Data collection process	11c	Describe planned method of extracting data from reports (such as piloting forms, done independently, in duplicate), any processes for obtaining and confirming data from investigators	X
Data items	12	List and define all variables for which data will be sought (such as PICO items, funding sources), any pre-planned data assumptions and simplifications	X
Outcomes and prioritization	13	List and define all outcomes for which data will be sought, including prioritization of main and additional outcomes, with rationale	X
Risk of bias in individual studies	14	Describe anticipated methods for assessing risk of bias of individual studies, including whether this will be done at the outcome or study level, or both; state how this information will be used in data synthesis	N/A
Data synthesis	15a	Describe criteria under which study data will be quantitatively synthesised	N/A
	15b	If data are appropriate for quantitative synthesis, describe planned summary measures, methods of handling data and methods of combining data from studies, including any planned exploration of consistency (such as I^2, Kendall's τ)	N/A
	15c	Describe any proposed additional analyses (such as sensitivity or subgroup analyses, meta-regression)	N/A
	15d	If quantitative synthesis is not appropriate, describe the type of summary planned	X
Meta-bias(es)	16	Specify any planned assessment of meta-bias(es) (such as publication bias across studies, selective reporting within studies)	N/A
Confidence in cumulative evidence	17	Describe how the strength of the body of evidence will be assessed (such as GRADE)	N/A

*** It is strongly recommended that this checklist be read in conjunction with the PRISMA-P Explanation and Elaboration (cite when available) for important clarification on the items. Amendments to a review protocol should be tracked and dated. The copyright for PRISMA-P (including checklist) is held by the PRISMA-P Group and is distributed under a Creative Commons Attribution Licence 4.0.**

From: Shamseer L, Moher D, Clarke M, Ghersi D, Liberati A, Petticrew M, Shekelle P, Stewart L, PRISMA-P Group. Preferred reporting items for systematic review and meta-analysis protocols (PRISMA-P) 2015: elaboration and explanation. BMJ. 2015 Jan 2;349(jan02 1):g7647.

For peer review only

BMJ Open

Upstream Interventions to Promote Oral Health and Reduce Socio-economic Oral Health Inequalities: A Scoping Review Protocol

Journal:	*BMJ Open*
Manuscript ID	bmjopen-2021-059441.R1
Article Type:	Protocol
Date Submitted by the Author:	26-Apr-2022
Complete List of Authors:	Dawson, Eleanor; University College London, Department of Epidemiology and Public Health<br>Stennett, Michelle; University College London, Department of Epidemiology and Public Health<br>Daly, Blánaid; Trinity College Dublin, Division of Public and Child Dental Health, Dublin Dental University Hospital and Dental School<br>Macpherson, Lorna; University of Glasgow<br>Cannon, Paul; University of Glasgow, University Library<br>Watt, Richard; University College London, Department of Epidemiology and Public Health
**Primary Subject Heading**:	Public health
Secondary Subject Heading:	Dentistry and oral medicine, Research methods
Keywords:	PUBLIC HEALTH, Health policy < HEALTH SERVICES ADMINISTRATION & MANAGEMENT, Protocols & guidelines < HEALTH SERVICES ADMINISTRATION & MANAGEMENT

Upstream Interventions to Promote Oral Health and Reduce Socio-economic Oral Health Inequalities: A Scoping Review Protocol

Word Count: 2120

Authors

Eleanor R. Dawson[1], Michelle Stennett[1], Professor Blánaid Daly[2], Professor Lorna M.D Macpherson[3], Paul Cannon[4] and Professor Richard G Watt[1]

1. Department of Epidemiology and Public Health, University College London, London, UK
2. Division of Public and Child Dental Health, Dublin Dental University Hospital and Dental School, University of Dublin, Trinity College Dublin, Dublin, Ireland
3. Dental Public Health Section, School of Medicine, Dentistry and Nursing, University of Glasgow, Glasgow, UK
4. University Library, Hillhead Street, University of Glasgow, G12 8QE, UK

Corresponding author: Eleanor Dawson; e.dawson@ucl.ac.uk

For peer review only

ABSTRACT

Introduction: Improving oral health and reducing oral health inequalities is an important global health priority. 'Upstream interventions' are a vital part of the collective effort to reduce oral disease burdens, however it is a rather nebulous term. Furthermore, there is little evidence on the effectiveness, impact and sustainability of upstream interventions that have focused on oral health and wider public health measures that impact on oral health. The aim of this scoping review is to systematically map and synthesize evidence on the effectiveness, impact and sustainability of upstream interventions on population oral health and reducing socio-economic oral health inequalities.

Methods and analysis: This scoping review will be conducted in accordance with the Joanna Briggs Institute (JBI) methodology and the Preferred Reporting Items for Systematic Reviews and Meta-Analyses Extension for Scoping Reviews (PRISMA-ScR) checklist. A detailed search strategy will be used to conduct a comprehensive search of electronic databases; Scopus, Embase and MEDLINE, PsycINFO and CINAHL, ASSIA and Cochrane Database of Systematic Reviews. A search of grey literature will also be completed to identify relevant dissertations, governmental reports and evaluations of implemented policies. Identification and extraction of data will be performed by two pairs of reviewers. Oversight and feedback will be provided by an independent expert advisory group.

Ethics and dissemination: This study will review published and available grey literature and does not require an ethics review. The scoping review protocol has been registered with the Open Science Framework. The final report will be circulated and disseminated through publication and feed into the work of the ongoing Lancet Commission on Oral Health. Due to the policy relevance of this work, discussions will take place with key stakeholders regarding the implications of the findings for future policy development.

Strengths and limitations of this study

- This scoping review will adopt a robust methodology in accordance with standard guidelines and checklists.
- The review aims to utilise seven electronic databases and a tailored search strategy to retrieve as many relevant published works as possible.
- The review will include literature published in any language and in the grey literature to create a comprehensive picture of upstream interventions.
- The review will assess the broad evidence base for upstream interventions on oral health outcomes and socio-economic inequalities, but will not undertake a detailed evaluation of interventions.

For peer review only

INTRODUCTION

Oral diseases are a highly prevalent global public health problem, affecting approximately 3.5 billion people, which equates to almost half of the world's population (1). Oral diseases (such as dental caries, periodontal disease, and oral cancer) are largely preventable and in recent decades some improvements in oral health have been observed predominantly in high-income countries (2). However dental caries in many low- and middle-income countries appears to be on the increase, linked to economic development and increasing availability and consumption of free sugars (3). Even in high-income countries with lower disease burdens, dental services are largely treatment oriented without fully addressing the underlying causes of oral diseases (4).

Poor oral health may lead to profound impacts on individuals and wider society. Pain or sepsis (4) lower self-esteem (5), reduced school attendance and poorer educational performance (6), poorer quality of life (7) and reduced work productivity (8) are all common impacts of oral diseases. There are also significant economic costs associated with dental treatment and lost productivity, amounting to approximately $544 billion globally in 2015 (9). Oral diseases disproportionately affect poorer, socially disadvantaged and marginalised groups; such differences are deemed to be avoidable, unfair and unjust in modern society (3). Furthermore, stark social gradients exist between socio-economic position and the prevalence and severity of oral conditions (10,11), reflecting similar patterns of inequality seen in general health.

Contemporary concepts regarding the determinants of health now acknowledge the underlying influence of structural and societal factors in socially patterning individual health behaviours and ultimately in determining disease levels (12). Indeed, McKinlay (13) originally utilised the metaphor of people being pushed into a flowing river to describe the causes, or upstream 'manufacturers' of ill health in comparison to the downstream causes of illness. However, this metaphor can also be employed to highlight the different intervention opportunities (upstream, midstream or downstream) for public health programmes and policies to improve population health (14).

Downstream interventions (individual/clinical/behavioural actions) have often been implemented to combat oral diseases and have failed to significantly reduce oral health inequalities or achieve sustainable improvements in oral health outcomes (15). For example, individual oral health education interventions tend to result in limited short-term positive changes in oral health related behaviours and health literacy, with marginal long-term improvements in clinical outcomes (16–18). Moreover, evidence has demonstrated that downstream interventions may adversely result in widening health inequalities between socio-economic groups, as they tend to provide more benefit for groups who are already advantaged (19). This widening of the health inequality gap is often referred to as the 'inverse prevention law' (20) and has been observed with oral health education interventions (21,22).

Upstream interventions are those that seek to address the underlying causative factors that lead to poor health, typically through action on broader structural, political, economic and environmental determinants. The term 'upstream intervention' is often a nebulous phrase to define in practice, as there may be difficulty in identifying broader public health approaches as purely 'upstream', as well as complexity in accurately defining population coverage and

For peer review only - http://bmjopen.bmj.com/site/about/guidelines.xhtml

effectiveness of interventions. Overall, upstream interventions attempt to achieve sustained health equity in a population, often outside of the sphere of health care systems (23). Upstream interventions generally aim to tackle broader social determinants (such as education, housing, access to safe drinking water and healthy food), or may create disincentives to engage in harmful health-related behaviours, such as fiscal policies to reduce consumption of sugary drinks (24). Upstream interventions are generally initiated by national governments or policy makers and operate in a top-down manner across whole populations.

A broad consensus now supports the need for a combination of downstream, midstream and upstream interventions in order to effectively prevent oral diseases and promote oral health equity across the population (4,25). However, questions remain over the extent of evidence surrounding the effectiveness of upstream interventions in promoting oral health and reducing socioeconomic health inequalities. This review will include the following upstream interventions for consideration; the implementation of water fluoridation schemes, regulations and fiscal measures aimed at reducing the consumption of tobacco, alcohol and sugar and interventions aimed at enhancing population welfare through improvement of housing, education and healthcare access. These upstream policies are those most commonly identified in the literature, however, the identification of other upstream interventions during the conduct of this review will also be included.

Although there are a wide range of persistent inequalities in oral health (such as inequalities between and within countries, ethnic differences and poorer outcomes in various vulnerable groups), this scoping review will focus on the impact of upstream interventions on promoting oral health and reducing socio-economic oral health inequalities.

For peer review only

For peer review only - http://bmjopen.bmj.com/site/about/guidelines.xhtml

METHODS

A scoping review has been chosen as the most appropriate form of evidence synthesis for this area of research as it will incorporate a broader scope and less restrictive inclusion criteria than a systematic review (26). The proposed work of this review will be linked to the ongoing Lancet Commission on Oral Health, which includes an oral health inequalities workstream, and the findings will be shared with other key stakeholders regarding the possible implications for policy development.

This scoping review will be conducted in line with the Joanna Briggs Institute (JBI) methodology (26) and the Preferred Reporting Items for Systematic Reviews and Meta-Analyses Extension for Scoping Reviews (PRISMA-ScR) checklist (27).

A preliminary search of SCOPUS, Google Scholar, Cochrane Database of Systematic Reviews and *JBI Evidence Synthesis* was conducted in June 2021. No ongoing or previously published scoping reviews on this topic were identified. Oversight and feedback of the methodology will be provided by an independent expert advisory group.

This scoping review study commenced in May 2021 with a planned completion date of July 2022.

Scoping review questions

The aim of this scoping review is to identify the current literature that documents implemented upstream interventions and their effect on promoting oral health and reducing socio-economic oral health inequalities. It will aim to address the following questions:

- What upstream interventions have been implemented to specifically target a reduction in socio-economic oral health inequalities and promote oral health?
- What relevant and related public health upstream interventions e.g., tobacco control policies have been implemented to reduce socio-economic health inequalities and promote health?
- Which upstream interventions have had an impact, are effective and are sustainable in reducing socio-economic oral health inequalities and improving population oral health?

Search Strategy

The search strategy will aim to retrieve both published studies and relevant grey literature. An initial limited search of SCOPUS and PubMed was undertaken to identify articles relevant to the topic. The text words contained in the titles and abstracts of relevant articles and the index terms used to describe the articles will be used to develop a full search strategy on: Scopus, Embase and MEDLINE (via Ovid), PsycINFO and CINAHL (via EBSCOhost), ASSIA (via ProQuest) and Cochrane Database of Systematic Reviews (via the Cochrane Library). Sources of grey literature to be searched include NTIS, NICE Evidence Search, TRIP, EThOS and WorldCat. The websites of relevant healthcare organisations will also be searched, for example the World Health Organization (WHO), Public Health England (PHE), World Bank and The Health Foundation. Relevant national or state level government policy

For peer review only - http://bmjopen.bmj.com/site/about/guidelines.xhtml

documents will be examined. A manual search of bibliographies of selected articles will be undertaken by the research team to extract any relevant literature or identify grey literature.

Given the difficulty defining upstream interventions, a multi-stranded search method (28) will be employed, with each strand representing an intervention of interest, namely: health inequalities, health disparities, health inequities, oral health promotion, tobacco control, sugar tax, upstream action, food advertising, food labelling and public health (Appendix A). The dental and oral health elements of the search will be adapted from the strategies employed by Waldron et al. (29) and Arora et al. (30). The sugar tax strand of the search will use the strategies of Pfinder et al. (31) and the equity-focused strand will use the strategies of Prady et al. (32).

Study selection

Inclusion criteria

-Participants and Context

Only studies that include population-wide polices or studies that target certain population groups will be included in this review. This includes populations that have been specifically targeted for the intervention or the general population. There is no minimum or maximum number for the size of the population group or age restrictions. The review will be limited to literature that includes implemented upstream interventions. The review will include all global settings where upstream interventions have been brought in to directly impact population oral health.

-Concepts and Definitions

The primary concept in this review is 'upstream interventions.' There is no unified definition of this term, but this scoping review has utilised a definition collating input from a variety of previous publications (13,14,15,25). Upstream interventions will be defined as 'strategies or policies that seek to address structural or environmental factors that impact population oral health and socio-economic oral health inequalities. This will include population level approaches, such as fiscal measures, regulation and legislation'.

Oral health inequalities are unequal and unfair differences in oral health outcomes across different groups in society (4). Such differences often disproportionally affect poorer and socially disadvantaged members of society. The association between poor oral health and socioeconomic status is well documented and is prevalent throughout the life course and across populations regardless of national income status.

-Sources and types of studies

This scoping review will include peer reviewed published journal articles, and grey literature including relevant government reports and policy documents. There is no limit on the date of publication. This study will include publications that have not been published in English and a complete translation will be sought. However, if it is not possible to obtain a full English translation the paper will be excluded and its omittance recorded in the final report.

For peer review only

For peer review only - http://bmjopen.bmj.com/site/about/guidelines.xhtml

and no language restrictions will be applied to the search. This review will consider a variety of study designs, including experimental, quasi-experimental, before and after studies and observational studies (including cohort studies, case-control studies and cross-sectional studies). In addition, systematic reviews will be included in this review, providing they meet the inclusion criteria. The review will also include other relevant public health policies and interventions, for example, sugar and tobacco control measures.

Exclusion criteria

Studies reporting on clinical interventions delivered in clinical settings and restricted to specific patient populations will be excluded from this review. Studies only reporting on oral health knowledge and attitudes and reviews of websites or industry documents will also not be included.

Patient and Public Involvement

This scoping review protocol was written with no patient or public involvement.

Data extraction

The final data from the search strategy will deposited into the database Rayyan (http://rayyan.qcri.org) where two pairs of reviewers will independently screen the titles and abstracts of each citation using the inclusion and exclusion criteria to identify eligible articles for full review. Full texts will be extracted into a data extraction form developed by the research team in Microsoft Word. The data extracted will include the following details: study characteristics (first author, publication year, country, published language, population size and characteristics, duration and setting) type of upstream intervention, outcomes and key findings relevant to the review question. The data extraction tool will be modified and revised as necessary during the process of extracting data from each included evidence source. If appropriate, authors of papers will be contacted to request missing or additional data, where required.

Collating, summarising and reporting the data

All of the relevant screened articles will be stored in the reference manager, Zotero (2020/ version 5.0.88). The summarised characteristics of the extracted studies will be presented visually in tabular form due to large number of studies that will be found. Results from the selected studies will also be presented in a narrative format which will relate directly to the review questions and aims.

ETHICS AND DISSEMINATION

The final protocol has been submitted for registration with Open Science Framework. A detailed final report will be produced on the review methods, results and recommendations following completion of the scoping review. A final paper presenting the review results will be also prepared for publication in a peer reviewed journal on completion of the review. The findings from this review will feed directly into the Lancet Commission on Oral Health and will be highlighted in the Commission Report depending upon the final structure of the report. Due to the strong policy relevance of this work, discussions will take place with the funder (The Borrow Foundation) on completion of the review to decide whether to hold a

For peer review only - http://bmjopen.bmj.com/site/about/guidelines.xhtml

dissemination workshop, as recommended by Levac et al (2010) (33) with key policy stakeholders regarding the relevance of the findings for future policy development. The findings from this review may also be presented through other relevant forums, such as international conferences or scientific meetings.

For peer review only

For peer review only - http://bmjopen.bmj.com/site/about/guidelines.xhtml

CONTRIBUTIONS

RGW, MS, BD and LM conceived the idea for the review. RGW, ED, MS, BD, PC and LM all contributed to the development of the protocol. ED, RGW and MS drafted the original manuscript. RGW, LM, PC and BD edited and revised the manuscript. ED conducted the preliminary search and PC provided expertise on the final search strategy. All authors edited and approved the final text prior to submission for publication.

ACKNOWLEDGMENTS

The research team would like to thank the expert advisory group for their input into the development of the protocol and the scoping review design and methodology. The advisory group is composed of, Professor Roger Keller Celeste (UFRGS, Brazil), Dr Manu Raj Mathur (University of Liverpool, UK/Public Health Foundation of India), Dr. Mary Jane McCallum (Canada), Professor Robert J. Weyant (University of Pittsburg, USA), Dr Jenny Godson and Dr Semina Makhani (Public Health England). The research team would also like to thank Sana Daniyal and Afshan Mirza for their support in undertaking the initial screening and reviewing of eligible papers.

FUNDING

This work was supported by The Borrow Foundation (UK Charity No: 1060308). The Borrow Foundation has no further role in the conduct of the review other than providing the designated funding.

COMPETING INTERESTS

None declared.

For peer review only

REFERENCES

1. Kassebaum NJ, Smith AGC, Bernabé E, et al. Global, Regional, and National Prevalence, Incidence, and Disability-Adjusted Life Years for Oral Conditions for 195 Countries, 1990-2015: A Systematic Analysis for the Global Burden of Diseases, Injuries, and Risk Factors. *J Dent Res* 2017;96(4):380-387.

2. GBD 2017 Oral Disorders Collaborators, Bernabe E, Marcenes W, Hernandez CR, et al. Global, Regional, and National Levels and Trends in Burden of Oral Conditions from 1990 to 2017: A Systematic Analysis for the Global Burden of Disease 2017 Study. *J Dent Res* 2020;99(4):362–73.

3. Watt RG, Daly B, Allison P, et al. Ending the neglect of global oral health: time for radical action. *The Lancet* 2019;394(10194):261–72.

4. Peres MA, Macpherson LMD, Weyant RJ, et al. Oral diseases: a global public health challenge. *The Lancet* 2019;20;394(10194):249–60.

5. Kaur P, Singh S, Mathur A, et al. Impact of Dental Disorders and its Influence on Self Esteem Levels among Adolescents. *J Clin Diagn Res* 2017;11(4):ZC05-ZC08

6. Rebelo MAB, Vieira JMR, Pereira JV, et al. Does oral health influence school performance and school attendance? A systematic review and meta-analysis. *Int J Paediatr Dent* 2019;29(2):138– 48.

7. Haag DG, Peres KG, Balasubramanian M, et al. Oral Conditions and Health-Related Quality of Life: A Systematic Review. *J Dent Res* 2017;96(8):864–74.

8. Kelekar U, Naavaal, S. Hours Lost to Planned and Unplanned Dental Visits Among US Adults. *Prev Chronic Dis* 2018;11;15:E05.

9. Righolt AJ, Jevdjevic M, Marcenes W, et al. Global, Regional, and Country-Level Economic Impacts of Dental Diseases in 2015. *J Dent Res* 2018;97(5):501–7.

10. Borrell LN, Crawford ND. Socioeconomic position indicators and periodontitis: examining the evidence. *Periodontol 2000* 2012;58(1):69–83.

11. Schwendicke F, Dörfer CE, Schlattmann P, et al. Socioeconomic inequality and caries: a systematic review and meta-analysis. *J Dent Res* 2015;94(1):10–8.

12. Solar O, Irwin A. *A Conceptual Framework for Action on the Social Determinants of Health*. WHO;2010.

13. McKinlay J. A case for refocusing upstream: the political economy of illness. In: Applying behavioral science to cardiovascular risk. Seattle: *American Heart Association* 1975:7–18.

14. McMahon NE. Framing action to reduce health inequalities: what is argued for through use of the 'upstream–downstream' metaphor? *J Public Health* Published Online First: 27 May 2021. doi: https://doi.org/10.1093/pubmed/fdab157

15. Watt RG. From victim blaming to upstream action: tackling the social determinants of oral health inequalities. *Community Dent Oral Epidemiol* 2007;35(1):1–11.

16. Sprod AJ, Anderson R, Treasure ET. Effective oral health promotion: literature review. In: Database of Abstracts of Reviews of Effects (DARE): Quality-assessed Reviews. York, UK: Centre for Reviews and Dissemination 1996.

For peer review only - http://bmjopen.bmj.com/site/about/guidelines.xhtml

17. Kay EJ, Locker D. Is dental health education effective? A systematic review of current evidence. *Community Dent Oral Epidemiol* 1996;24(4):231–5.

18. Watt RG, Marinho VC. Does oral health promotion improve oral hygiene and gingival health? *Periodontol 2000*. 2005;37:35–47.

19. Lorenc T, Petticrew M, Welch V, et al. What types of interventions generate inequalities? Evidence from systematic reviews. *J Epidemiol Community Health* 2013;67(2):190–3.

20. Gordon D, Shaw M, Dorling D, et al. Inequalities in health: Evidence presented to the independent inquiry into inequalities in health. Bristol, UK: Policy Press 1999.

21. Schou L, Wight C. Does dental health education affect inequalities in dental health? *Community Dent Health* 1994;11(2):97–100.

22. Qadri G, Alkilzy M, Franze M, et al. School-based oral health education increases caries inequalities. *Community Dent Health* 2018;30:35(3):153–9.

23. Williams DR, Costa MV, Odunlami AO, et al. Moving Upstream: How Interventions that Address the Social Determinants of Health can Improve Health and Reduce Disparities. *J Public Health Manag Pract* 2008;14(Suppl):S8-17.

24. World Health Organisation (WHO). *Taxes on sugary drinks: Why do it?* Geneva: World Health Organisation; 2017.

25. Baelum V. Dentistry and population approaches for preventing dental diseases. *J Dent*. 2011;39(Suppl 2):S9-19.

26. Aromataris E, Munn Z. JBI Manual for Evidence Synthesis. Australia: JBI, 2020.

27. Tricco AC, Lillie E, Zarin W, et al. PRISMA Extension for Scoping Reviews (PRISMAScR): Checklist and Explanation. *Ann Intern Med* 2018;169:467–473

28. National Institute of Health and Care Excellence (NICE). *Developing NICE guidelines: the manual*. NICE. 2020.

29. Waldron C, Nunn J, Phadraig CMG, et al. Oral hygiene interventions for people with intellectual disabilities. *Cochrane Database Syst Rev* 2019;31;5(5):CD012628.

30. Arora A, Khattri S, Ismail NM, et al. School dental screening programmes for oral health. *Cochrane Database Syst Rev* 2019;8;(8):CD012595.

31. Pfinder M, Heise TL, Boon MH, et al. Taxation of unprocessed sugar or sugar-added foods for reducing their consumption and preventing obesity or other adverse health outcomes. *Cochrane Database Syst Rev* Published Online First: 9 April 2020. doi: 10.1002/14651858.CD012333.pub2.

32. Prady SL, Uphoff EP, Power M, et al. Development and validation of a search filter to identify equity-focused studies: reducing the number needed to screen. *BMC Med Res Methodo* 2018;18(106).

33. Levac D, Colquhoun H, O'Brien KK. Scoping studies: advancing the methodology. *Implementation Science* 2010;5:69.

For peer review only

APPENDIX

Appendix A: Search strategy for a scoping review protocol on upstream interventions to promote oral health and reduce socio-economic oral health inequalities.

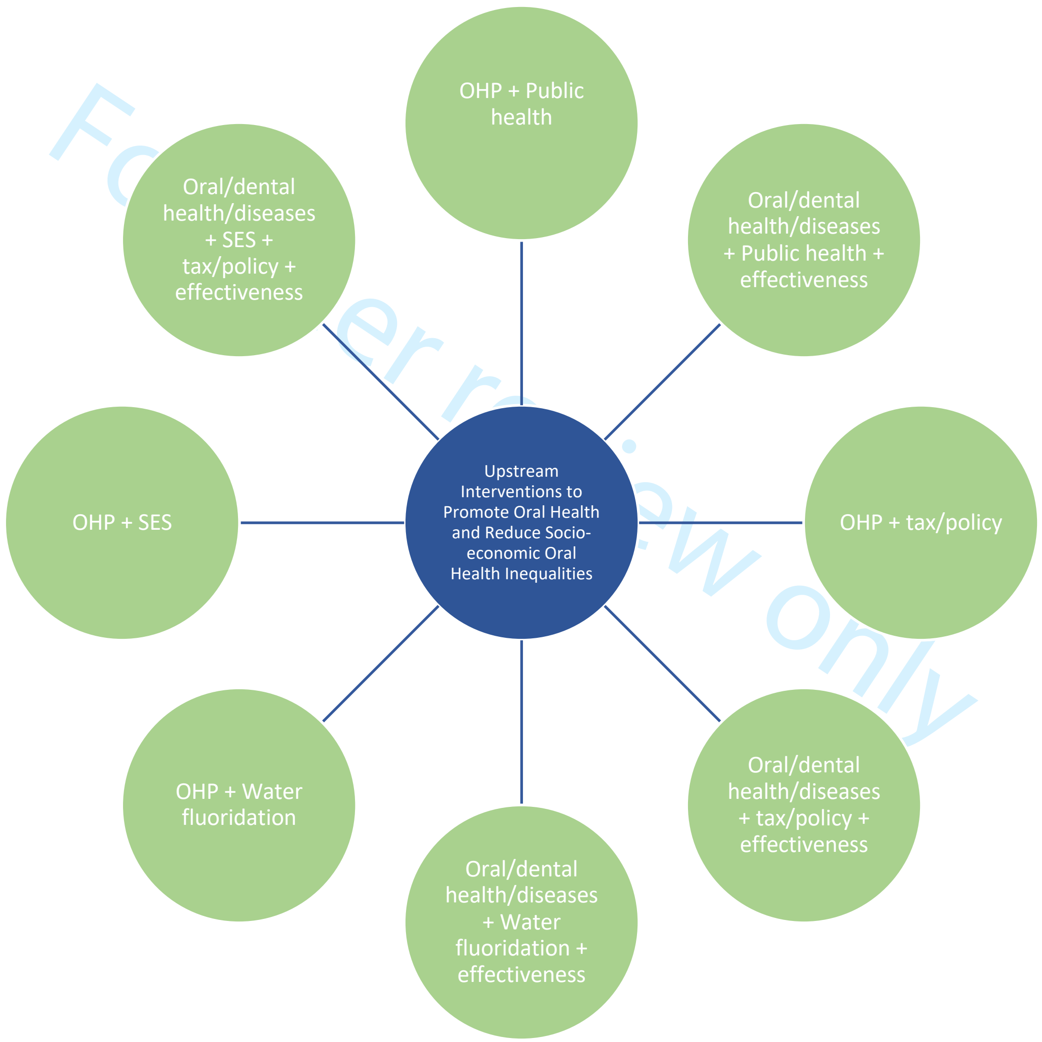

PRISMA-P (Preferred Reporting Items for Systematic review and Meta-Analysis Protocols) 2015 checklist: Upstream Interventions to Promote Oral Health and Reduce Socio-economic Oral Health Inequalities: A Scoping Review Protocol

Section and topic	Item No	Checklist item	Completed
**ADMINISTRATIVE INFORMATION**			
Title:			
Identification	1a	Identify the report as a protocol of a systematic review	X
Update	1b	If the protocol is for an update of a previous systematic review, identify as such	N/A
Registration	2	If registered, provide the name of the registry (such as PROSPERO) and registration number	X
Authors:			
Contact	3a	Provide name, institutional affiliation, e-mail address of all protocol authors; provide physical mailing address of corresponding author	X
Contributions	3b	Describe contributions of protocol authors and identify the guarantor of the review	X
Amendments	4	If the protocol represents an amendment of a previously completed or published protocol, identify as such and list changes; otherwise, state plan for documenting important protocol amendments	N/A
Support:			
Sources	5a	Indicate sources of financial or other support for the review	X
Sponsor	5b	Provide name for the review funder and/or sponsor	X
Role of sponsor or funder	5c	Describe roles of funder(s), sponsor(s), and/or institution(s), if any, in developing the protocol	X
**INTRODUCTION**			
Rationale	6	Describe the rationale for the review in the context of what is already known	X
Objectives	7	Provide an explicit statement of the question(s) the review will address with reference to participants, interventions, comparators, and outcomes (PICO)	X
**METHODS**			
Eligibility	8	Specify the study characteristics (such as PICO, study design, setting, time frame) and report characteristics (such as years	X

For peer review only

criteria		considered, language, publication status) to be used as criteria for eligibility for the review	
Information sources	9	Describe all intended information sources (such as electronic databases, contact with study authors, trial registers or other grey literature sources) with planned dates of coverage	X
Search strategy	10	Present draft of search strategy to be used for at least one electronic database, including planned limits, such that it could be repeated	X
Study records:			
Data management	11a	Describe the mechanism(s) that will be used to manage records and data throughout the review	X
Selection process	11b	State the process that will be used for selecting studies (such as two independent reviewers) through each phase of the review (that is, screening, eligibility and inclusion in meta-analysis)	X
Data collection process	11c	Describe planned method of extracting data from reports (such as piloting forms, done independently, in duplicate), any processes for obtaining and confirming data from investigators	X
Data items	12	List and define all variables for which data will be sought (such as PICO items, funding sources), any pre-planned data assumptions and simplifications	X
Outcomes and prioritization	13	List and define all outcomes for which data will be sought, including prioritization of main and additional outcomes, with rationale	X
Risk of bias in individual studies	14	Describe anticipated methods for assessing risk of bias of individual studies, including whether this will be done at the outcome or study level, or both; state how this information will be used in data synthesis	N/A
Data synthesis	15a	Describe criteria under which study data will be quantitatively synthesised	N/A
	15b	If data are appropriate for quantitative synthesis, describe planned summary measures, methods of handling data and methods of combining data from studies, including any planned exploration of consistency (such as I^2, Kendall's τ)	N/A
	15c	Describe any proposed additional analyses (such as sensitivity or subgroup analyses, meta-regression)	N/A
	15d	If quantitative synthesis is not appropriate, describe the type of summary planned	X
Meta-bias(es)	16	Specify any planned assessment of meta-bias(es) (such as publication bias across studies, selective reporting within studies)	N/A
Confidence in cumulative evidence	17	Describe how the strength of the body of evidence will be assessed (such as GRADE)	N/A

*** It is strongly recommended that this checklist be read in conjunction with the PRISMA-P Explanation and Elaboration (cite when available) for important clarification on the items. Amendments to a review protocol should be tracked and dated.** **The copyright for PRISMA-P (including checklist) is held by the PRISMA-P Group and is distributed under a Creative Commons Attribution Licence 4.0.**

From: Shamseer L, Moher D, Clarke M, Ghersi D, Liberati A, Petticrew M, Shekelle P, Stewart L, PRISMA-P Group. Preferred reporting items for systematic review and meta-analysis protocols (PRISMA-P) 2015: elaboration and explanation. BMJ. 2015 Jan 2;349(jan02 1):g7647.

For peer review only

For peer review only - http://bmjopen.bmj.com/site/about/guidelines.xhtml
